# Supplementary material for: A larger augmented-reality field of view improves interaction with nearby holographic objects
Source: PLoS One. 2024 Oct 21;19(10):e0311804. doi: 10.1371/journal.pone.0311804 (PMC11493412; doi:10.1371/journal.pone.0311804)
Supplement: Attachment — Submitted filename: Response to Reviewers.docx [file pone.0311804.s002.docx]

Dear professor Cannavò,

Thank you very much for your interest in our manuscript titled "A greater augmented-reality field of view improves interaction with nearby holographic objects." We appreciate the thoughtful comments of the reviewers and we have revised our manuscript accordingly. In brief, we have included an extra analysis of the effect of differences in AR field of view on gait speed and we have added a detailed overview with specifications of HoloLens 1 and 2. Changes have been highlighted in the copy of the revised manuscript and are also specified below. As requested, we have also uploaded a clean copy.

We hope that our manuscript is now suitable for publication in PLOS ONE.

Sincerely, on behalf of my co-authors,

Eva Hoogendoorn

Our point-by-point response:

Reviewer 1

1. **The difference in FOVs in this study is small. The reviewer also considers that only two conditions are really limited.**

Response: We do acknowledge that it would be interesting to include more conditions with larger differences in AR-FOV. To do so, VST-HMDs are required. Our study, however, focused on OST-HMDs, see also our response on comment 2 of reviewer 1. We included this relevant comment as a recommendation for future research in the Discussion section:

*“Alternatively, one could consider including a video see-though device (e.g., Apple vision Pro) instead of only optical see-through devices used in the current study, allowing for a more fine grained and wider evaluations of the effect of AR-FOV size on head orientation and gait speed for interacting with nearby AR content.”*

2. **There are OST-HMD and VST-HMD. The authors had better mention more conclusive MR-based gait modules. (but optional, not necessary).**

Thank you for this relevant comment. Our research only included OST-HMDs. Based on the reviewers' comment, we expressed this more clearly in the revised manuscript, see Table 1 in the Methods section for a detailed overview of specifications of the HMDs. We are particularly interested in OST-HMDs, because of their potential for gait assistance, gait modulation, gait-and-balance assessment and gait-and-balance training (as was already described in the Introduction). We do agree that VST-HMDs are interesting to include in future research and we adjusted the text accordingly as specified in our response to your comment 1.

3. **Citing and discussing depth perception study might be helpful.**

Response: The reviewer rightly mentioned the importance of depth perception. Since we added problems with depth perception in the exclusion criteria (see also our response to your comment 6), a discussion on depth perception was not included.

4. **The objectives should be mentioned more clearly.**

Response: We have revisited our objectives, amended them in light of the reviewers’ comments to examine the effect of AR-FOV on gait characteristics (see also our response to your comment 9 below) and adjusted them for the sake of clarity. The objectives now read as follows (see Introduction section):

*“The primary objective of this study was to systematically evaluate the effect of AR-FOV size on the head orientation required for viewing and interacting with floor-based real-world and holographic objects. The secondary objective was to evaluate the effect of AR-FOV size on gait speed when interacting with floor-based real-world and holographic objects.”*

5. **How to make the research question is unclear. The review considers that it is too natural that higher FOV obviously leads to the higher number of obstacles that people can recognize. What elements are unknown and how to investigate them should be explained more clearly.**

Response: We agree with the reviewer that it is rather obvious that a larger AR-FOV size leads to a higher number of visible AR objects. Therefore, we decided to move this finding (original Experiment 1) to the Methods section and presented this as a methodological validation of hardware specifications (see also our response to comment 2 of reviewer 2):

*“To methodologically validate if participants perceived differences in the AR-FOV size, we quantified the number of visible real-world and holographic objects reported by our participants while wearing HoloLens 1 and 2. As expected, participants reported more real-world than holographic objects because the AR-FOV size is smaller than one’s own unobstructed FOV size (210º×150º [25]). However, the difference between the reported real-world and holographic objects was significantly smaller for HoloLens 2 because its AR-FOV size is greater than that of HoloLens 1 (Figs 1A-B).”*

However, it still remains unclear what the effect of the AR-FOV size is on human perceptual-motor behavior in terms of head orientation required to interact with the presented AR content as well as the resultant gait speed during these interactions, as evaluated in this article. We therefore specified this as our objectives (see also our response to your comment 4 above).

6. **How was depth perception of the participants.**

Response: The reviewer rightly hinted on the importance of depth perception of the participants. Two persons that had problems with depth perception were excluded from participating. We specified this as follows in the Methods section of this revision: *“Participants had no conditions interfering with gait function and no restrictions interfering with their depth perception or vision (after potential corrective aids).”*

7. **How to quantify the head orientation?**

Response: Head orientation was derived from the AR-headset orientation data, normalized to baseline orientation (i.e., to correct for potential between-headset-condition differences in positioning of the AR visor in front of participants’ eyes), as described in detail in the Methods section. We have expanded the explanation to improve visibility and clarity in the text:

*“Headset position and orientation data were recorded with either HoloLens 1 or 2 with a sampling frequency of 30 Hz to analyze participants’ head position along the line of progression on the walkway (i.e., increasing along the walkway from the starting position) and head orientation in terms of pitch (i.e., rotation of the headset along the lateral axis such that a downward rotation corresponds to an increase in the orientation angle).”*

We also added a short explanation within the description of figure 3: *“Head orientation was derived from the AR-headset orientation data, normalized to the baseline head orientation.”*

8. **Why was the closest condition 75cm along the walkway?**

Response: this was a somewhat arbitrary choice, thought to resemble a natural initiation step length of 60cm (i.e., 75 cm refers to the center of the 30cm stepping target).

9. **Walking velocity should be reported because it affects a human vision, such as optical flow, and preparation time for stepping obstacles.**

Response: Adding gait speed is an excellent suggestion, as indeed this could have been affected by the AR-FOV size and associated changes in head orientation required to interact with AR content. We could derive gait speeds from the AR positional headset data. We have included gait speed in the revised manuscript as a secondary objective of the study. The effects, if any, were small in magnitude but significant for some conditions, with slightly slower gait speeds (~8.8 cm/s) for interacting with floor-based holographic than real-world content.

10. **Please add statistical power.**

Response: From a statistical point of view, it is unusual to add a power-analysis post-hoc. However, we do report the effect size (as partial eta squared) so that future studies are in a better position to perform an a-priori power analysis.

11. **In this study, the OST-HMD was used for investigation. However, when it comes to investigation of the FOV effects, VST-HMD is useful to make the conditions of the FOV. The reviewer hopes to see insights of meaning of using OST-HMD for investigation of the FOV effects.**

Response: Thank you for this relevant comment. We are particularly interested in OST-HMDs, because of its potential for gait assistance, gait modulation, gait-and-balance assessment and gait-and-balance training in real-world settings (as described in the Introduction, see also our response to your comment 2). With OST-HDMs, system errors or empty battery impact the AR content, but not the visibility of the real world. This is, however, different with VST-HMDs. We therefore focused on OST-HMDs that are available today. We included an explanation in the Discussion section:

*“ Alternatively, one could consider including a video see-though device (e.g., Apple vision Pro) instead of only optical see-through devices as used in the current study, allowing for more fine grained and wider evaluations of the effect of AR-FOV size on head orientation and gait speed for interacting with nearby AR content. Video see-through devices, however, seem less suitable for assisting and modulating gait in health-related fields, mainly because of the risk of losing the vision of the real environment in case of system errors or an empty battery, which could lead to dangerous situations in daily life as wearers then find themselves in a pitch-black environment.”*

Note: since recent studies with OST-HMDs more often refer to the technology as augmented-reality instead of mixed-reality, we decided to change the term ‘mixed-reality (MR)’ into ‘augmented-reality (AR)’ throughout the manuscript.

12. **Some significant differences were found between Hololens 1 and Hololens 2. But how this differences affect human motion is unclear. The reviewer wants to encourage this analysis (literature based discussion is possible).**

Response: Thank you for this excellent suggestion, which we addressed by including gait speed as a secondary objective, as detailed in our response to your comment 9.

13. **The effect of downward head orientation on human cognition also might be able to be discussed.**

Response: This is an interesting comment, but it lies outside the scope of this study.

Reviewer 2

1. **The Authors analyzed the influence of the Mixed Reality (MR) field of view in the case of users suffering from Parkinson’s disease. The head orientations required for viewing and interacting with real-world and holographic floor-based objects during standstill and walking conditions were studied. From a statistical point of view, the study sample is too small to draw any reliable conclusions.**

Our study sample size of 16 participants is a convenience sample, which allowed for a fully block-randomized counterbalancing of the order of the experimental conditions, suitably large for a repeated-measures design wherein each participant performs all conditions.

We would like to emphasize that our study is a technical validation study executed with healthy adults (and not with people with Parkinson’s disease like reviewer 2 suggested). It is not unusual to include 10-20 participants in technical validation studies as has been done in relevant previous studies [1-3], to name a few. Furthermore, we implemented a repeated-measure design and executed all walking trials three times, to increase the reliability of our results. We are therefore confident that our sample size of n=16 was sufficient for this study, as corroborated by profound effects with large effects sizes in line with our expectations.

2. **Some of the observations from the experiments are derived from the technical specifications of the hardware (differences between HoloLens 1 and HoloLens 2). In the context of device specifications, it is not clear what the new paper brings.**

Response: We agree with the reviewer that the observations from Experiment 1 logically follow from differences in technical specifications between the HoloLenses. As detailed in our response to the related comment 5 of Reviewer 1, we decided to move this finding (original Experiment 1) to the Methods section and presented this as a methodological validation of hardware specifications, which are now also detailed for HoloLens 1 and 2 in the new Table 1.

*“To methodologically validate if participants perceived differences in the AR-FOV size, we quantified the number of visible real-world and holographic objects reported by our participants while wearing HoloLens 1 and 2. As expected, participants reported more real-world than holographic objects because the AR-FOV size is smaller than one’s own unobstructed FOV size (210º×150º [25]). However, the difference between the reported real-world and holographic objects was significantly smaller for HoloLens 2 because its AR-FOV size is greater than that of HoloLens 1 (Figs 1A-B).”*

However, it still remains unclear what the effect of the AR-FOV size is on human perceptual-motor behavior in terms of head orientation required to interact with the presented AR content as well as the resultant gait speed during these interactions, as evaluated in this article.

3. **The English language requires correction.**

Response: Point well taken. We made several textual corrections throughout the manuscript.

4. **The included figures are of very low quality.**

Response: It remains unclear what the reviewer meant with very low quality. The figure files are uploaded in the Preflight Analysis and Conversion Engine (PACE) digital diagnostic tool to ensure that figures meet PLOS requirements.

**References**

1.	Binaee K, Diaz GJ. Assessment of an augmented reality apparatus for the study of visually guided walking and obstacle crossing. Behavior Research Methods. 2019;51(2):523-31. doi: 10.3758/s13428-018-1105-9.

2.	Chan ZYS, MacPhail AJC, Au IPH, Zhang JH, Lam BMF, Ferber R, Cheung RTH. Walking with head-mounted virtual and augmented reality devices: Effects on position control and gait biomechanics. PLOS ONE. 2019;14(12):e0225972. doi: 10.1371/journal.pone.0225972.

3.	Koop MM, Rosenfeldt AB, Johnston JD, Streicher MC, Qu J, Alberts JL. The HoloLens Augmented Reality System Provides Valid Measures of Gait Performance in Healthy Adults. IEEE Transactions on Human-Machine Systems. 2020;50(6):584-92. doi: 10.1109/THMS.2020.3016082.
