# Supplementary material for: A larger augmented-reality field of view improves interaction with nearby holographic objects
Source: PLoS One. 2024 Oct 21;19(10):e0311804. doi: 10.1371/journal.pone.0311804 (PMC11493412; doi:10.1371/journal.pone.0311804)
Supplement: Attachment — Submitted filename: Response to Reviewers.docx [file pone.0311804.s003.docx]

Dear professor Cannavò,

Thank you for your editorial feedback and for the time you have taken to review our manuscript. We appreciate the positive feedback from Reviewer 1 who recommended to accept our manuscript for publication in PLOS ONE.

We also fully understand that you have to take Reviewer 2's concerns seriously. However, we respectfully disagree with Reviewer 2's assessment. He or she clearly misread (or even did not fully read) our manuscript: there was no between-groups comparison in our manuscript, contrary to what was suggested by the reviewer. Our study is a technical validation study conducted with 16 healthy adults (not persons with Parkinson’s disease as again was misread by the same Reviewer in the initial round of reviews). All participants performed all headset [HoloLens 1, HoloLens 2] by content [real-world cues, AR cues] conditions in a fully counter-balanced within-subject repeated-measures design, which was also addressed in our previous response letter to a similar comment of Reviewer 2. Reviewer 2 further mentioned that there was an "ignorance of the nomenclature or specifics", which we believe is not accurate, as explained in more detail in the point-by-point response below. In brief, Mixed Reality (MR, where the Reviewer is clearly a fan of) is the umbrella term used for all realities within Milgram’s famous virtuality spectrum ranging from Reality to Virtual Reality, where closer to Reality the term Augmented Reality (AR, which we used in our manuscript) is pertinent while closer to Virtual Reality (VR) the term Augmented Virtuality (AV) is pertinent. Hence, both the use of AR and MR terminology would be accurate for our manuscript, with AR being more specific than MR in that regard as we augment the real world with holographic cues (AR, closer to the Reality than to the Virtual Reality endpoint of Milgram’s virtuality spectrum).

Besides that, Reviewer 2 stated that the obtained results are an obvious consequence of the different technical parameters of HoloLens 1 and 2, thereby implicitly evaluating our manuscript on perceived significance. We believe that this interpretation reflects a personal preference (similar to the MR vs. AR choice), not meeting the criteria set forth by PLOS ONE. As PLOS ONE evaluates research based on scientific validity, robust methodology, and high ethical standards—rather than perceived significance—we aimed to provide a comprehensive and methodologically sound evaluation of how differences in technical parameters between the two HoloLens generations affected perception of holographic content as well as the interaction with them in terms of gait speed and the required head orientations to pick up task-relevant AR and real-world information. Our study offers detailed insights in those regards that are not immediately apparent without scientific investigation. Therefore, we believe that our research meets the criteria for publication ([Journal Information | PLOS ONE](https://journals.plos.org/plosone/s/journal-information#loc-criteria-for-publication)).

Given these points, we kindly request to respectfully reweigh Reviewer 2’s concerns (which are factual non-issues given the publication criteria of PLOS ONE) and to reconsider our revised manuscript for publication in PLOS ONE.

Sincerely, on behalf of my co-authors,

Eva Hoogendoorn

Our point-by-point response:

Reviewer 1
**I have checked the all answerers and modifications.
All my comments have been addressed.**

Thank you very much for reviewing our manuscript and endorsing it for publication.

Reviewer 2

1. **The Authors do not analyze Parkinson's disease, so they should remove references or any mention to it in the abstract and introduction.**

Response: Thank you for your feedback. We have removed the reference to Parkinson's disease from the abstract. However, we have retained the reference to Parkinson’s disease in the introduction to provide essential context regarding the broader application of AR technology in general, and the significance of AR cueing applications in specific.

2. **The Authors analyze/perform experiments in the area of technical properties/features of the Mixed Reality equipment itself. The added value of the paper is not clear (the obtained results are an obvious consequence of the technical parameters of the HoloLens generation).**

Response: While we understand and respect your perspective, we believe this interpretation of (presumably) the difference in the AR/MR field of view size between the two HoloLens generations may be more reflective of your perceived significance of our work than being ground for rejection of our paper given the publications criteria set forth by PLOS ONE.

That is, the journal's focus on "quality-based selection" emphasizes the importance of scientific validity, robust methodology, and high ethical standards over perceived significance. Our research is designed to adhere to these principles. Specifically, we aimed to provide a comprehensive and methodologically sound evaluation of how differences in technical parameters (FOV-size) between the two HoloLens generations affected the perception and pick-up of holographic information (in comparison to real-world information) as well as the interaction with this information in terms of gait speed and the required head orientations to pick up task-relevant AR and real-world information, offering insights that are not immediately apparent without scientific investigation. Whether this is perceived as a significant contribution to science or not is at best a subjective interpretation, but not a PLOS ONE publication criterium.

3. **Conducting statistical research on 16 cases divided into groups (10 individuals versus 6 individuals) does not provide reliable conclusions.**

Response: We are afraid that you misread our study: there was no division of groups. Our study is a technical validation study conducted with 16 healthy adults, all performing all four headset (HoloLens 1 and 2) by content (real-world and holographic) conditions in a fully counter-balanced within-subject repeated-measures design.

We would like to point out that this was already addressed in our previous response letter:

*“We would like to emphasize that our study is a technical validation study executed with healthy adults (and not with people with Parkinson’s disease like reviewer 2 suggested). It is not unusual to include 10-20 participants in technical validation studies as has been done in relevant previous studies [1-3], to name a few. Furthermore, we implemented a repeated-measures design and executed all walking trials three times, to increase the reliability of our results. We are therefore confident that our sample size of n=16 was sufficient for this study, as corroborated by profound effects, in line with our expectations, with large effect sizes.”*

4. **The title concerns Augmented Reality as well as main text and the Authors investigate Mixed Reality. Which indicates their ignorance of the nomenclature or specifics of the equipment.**

Response: We feel that this comment is not only offensive but also inaccurate. As frontrunners in the field, we are well aware of nomenclature and of the importance of its precise use in light of Milgram’s virtuality continuum, ranging from Reality (real environment) on the one end to Virtual Reality (virtual environment) on the other end of the continuum. Within these extremes, we find ourselves in a mixed reality (MR), a rather broad and unspecific umbrella term covering all realities except pure Reality and pure Virtual Reality. To be more precise, mixed-realities that are closer to the Reality end than to the Virtual Reality end of the continuum have been coined Augmented Reality (AR) while mixed-realities closer to the Virtual Reality than to the Reality end have been coined Augmented Virtuality. Clearly, our study, in which we augmented the real world with holographic cues, represents Augmented Reality.

*Milgram, P., and Kishino, F. (1994). A taxonomy of mixed reality visual displays. IEICE Trans. Inform. Syst. 77, 1321–1329.*
